# Factors Associated with Fatigue among Men Aged 45 and Older: A Cross-Sectional Study

**DOI:** 10.3390/ijerph120910897

**Published:** 2015-09-02

**Authors:** Wei-Quan Lin, Meng-Juan Jing, Jie Tang, Jia-Ji Wang, Hui-Shan Zhang, Le-Xin Yuan, Pei-Xi Wang

**Affiliations:** 1 Department of Preventive Medicine, School of Public Health, Guangzhou Medical University, Guangzhou, 510182, China; E-Mails: linweiquan0503@163.com (W.-Q.L.); gytanjie@163.com (J.T.); wjiaji@163.com (J.-J.W.); zhanghuishan_25@163.com (H.-S.Z.); 2 Institute of Public Health, School of Nursing, Henan University, Kaifeng, 475004, China; E-Mail: jing53905@163.com; 3 Department of Nursing, School of Nursing, Guangzhou Medical University, Guangzhou, 510182, China; E-Mail: yuanlexin2015@163.com

**Keywords:** fatigue, middle-aged and older men, prevalence, cross-sectional study

## Abstract

**Background and Purpose**: Fatigue is one of the most common symptoms reported in several studies; but few studies have concentrated on the male population, especially for the middle-aged and older men who are exposed to greater fatigue risk. The purpose of this study was to explore the prevalence of fatigue and identify the risk factors of fatigue among men aged 45 and older in China. **Methods**: This study was part of a cross-sectional study on community health in Shunde (Guangdong Province, China). A total sample of 1158 men aged 45 and older were included. Sociodemographic characteristics, health and lifestyle factors and the Chalder Fatigue Scale (CFS) were measured by structured questionnaires through face-to-face interviews. Multivariate logistic regression was applied to determine the risk factors of fatigue. **Results:** Approximately 30% of participants experienced fatigue. Older age (≥75 years: adjusted OR 3.88, 95% CI 2.09–7.18), single marital status (1.94, 1.04–3.62), unemployed status (1.68, 1.16–2.43), number of self-reported chronic diseases (≥2 chronic diseases: 2.83, 1.86–4.31), number of individuals’ children (≥4 children: 2.35, 1.33–4.15), hospitalization in the last year (1.61, 1.03–2.52) were all significantly associated with increased risk of fatigue, while regular exercise (0.46, 0.32–0.65) was a protective factor against fatigue. *Conclusions*: Fatigue was usual in males and several factors were associated with the fatigue. These findings may have implication in risk assessment of fatigue and help in developing and implementing targeted interventions in middle-aged and elderly males.

## 1. Introduction

Fatigue is a non-specific symptom but one of the most common ones reported in several studies [1,2]. It is defined as one’s state of overwhelming, debilitating, sustained exhaustion and decreased ability to perform daily activities, and that cannot be relieved by rest [3].

The men aged 45 and older are more susceptible to various kinds of chronic disease, such as hypertension, diabetes [4] and mental disorders, like depression and anxiety [5], which may be related to fatigue. Additionally, men would lose almost twice muscle strength than women with aging, which may also be an increased risk of fatigue [6]. What’s more, great pressure caused by rapid economic and social transformations may be another reason for fatigue [7]. Previously, our research team completed research on fatigue among the female population [8]. However, middle-aged and older men are also exposed to great risk of fatigue, which we should not ignore.

Fatigue also has significant consequences for society. Several studies have showed that fatigue sustained for a long time can predict future morbidity and mortality [9]. Fatigue also can result in declines in worker productivity due to the debilitating nature of fatigue [10]. It is estimated that annually $ 8,554 are lost in household earnings in Georgia [11] and in the whole United States the cost of declines in household/labor productivity amounts to $ 9.1 billion per year [12]. It may also impair individuals’ quality of life [13]. Therefore, fatigue is an outstanding public health issue that requires the development and implementation of effective interventions and more studies to be conducted to understand fatigue and its risk factors.

Previous studies, both abroad and domestic, have shown that the prevalence of fatigue among the general population varied between 2.36% and 75.7% [14,15,16,17,18,19]. Among males, the incidence of fatigue ranged from 1.2% to 34.8% [15,17,18,19,20,21]. Peng and colleagues even reported rates of fatigue of up to 81.8% among male workers in China [22]. The wide variations in fatigue prevalence in those studies may be related to the definition of fatigue used (less or more restrictive standardization; different in duration), the assessment methods (conducted by physician or based on self-reporting), the measurement instruments, racial differences [23], and so on. Nonetheless, it is indisputable that fatigue is a widely experienced symptom in the general population.

Some potential risk factors of fatigue were demonstrated in previous studies. Junghaenel and colleagues confirmed that being female, in an older age group, with unmarried status and lower education level were associated with increased fatigue [24], which was consistent with studies from China [20,22,25]. Wong and Fielding’s research found that fatigue was associated with long-term health problems, mental disease (depression, anxiety) and low socioeconomic status [15], concomitantly, Yao and colleagues also supported those findings with data from a study among the general population in Daqing, China [16]. Other studies revealed that unhealthy diets, work duration and sleep problems were related to fatigue [25,26], while it is uncertain whether other factors, such as hospitalization in the last year, living arrangements and the individuals’ number of children, may affect fatigue in men. Specifically, there are specific studies about the epidemiology and factors associated with fatigue among children [27], adolescents [28,29], the elderly and females [30,31], but limited studies have been conducted to investigate the epidemiology and fatigue risk factors among males, and to the best of our knowledge, there has been no special research on middle-aged and elderly males in China.

We therefore set out to investigate a specific study object: men (≥45 years), and fatigue was defined at least a one-time feeling of unexplained fatigue in the last month. The purpose of the present study was: (1) to assess the prevalence of fatigue; (2) to determine the factors associated with fatigue in men aged 45 years and older.

## 2. Methods

### 2.1. Respondents and Procedures

In July 2014, a cross-sectional study on community health was conducted in the Shunde municipality of Guangdong Province in China. A simple random sampling method was employed to generate the study sample. Five percent of the total households in this municipality were selected randomly, and all the family members were eligible for inclusion in the present study. A total of 2080 households, including 6802 individuals, were selected as the study participants. However, there were 243 individuals who refused to participate (those who didn’t cooperate with the investigation or respond after three home visits were regarded as refusers and were excluded in this study), therefore, 6559 residents were finally included in the study. In the present study, we only selected the data of men aged 45 years and older from the data of all participants, excluding those who had severe mental disorders (those who were incapable of completing Chalder fatigue scale due to insanity, such as acute phase schizophrenia), so the study subject comprised a total sample of 1158 males.

The face-to face interviews were conducted by a group composed of a physician or a nurse (local healthcare staff) and at least two medical students. All students in the group were trained before the investigation in order to standardize the survey technique. The interviews took place in the participants’ homes and data were collected by using structured study questionnaires. The survey took about 20–30 min to complete for each participant. It was noteworthy that we arranged a supervisor to each group to ensure the investigation were properly conducted.

### 2.2. Instruments

#### 2.2.1. Assessment of Fatigue

In this study, the Chalder fatigue scale (CFS) was used to assess fatigue. It included 11 items [32,33]. The scale yielded two dimensions: physical fatigue (PF, including seven items) and mental fatigue (MF, including four items). Each item was scored 0 or 1, and the total scores ranged from 0 to 11, with a higher score indicating a higher degree of fatigue, as in addition a cut-off score of 4 or more was used to define fatigue cases [15]. Furthermore, the CFS is considered culturally sensitive and has no significant respondent burden, it provides fatigue measurements with good reliability and validity and it has also been widely used in both the general and clinical populations in China [34,35].

#### 2.2.2. Health and Lifestyle Factors

The health and lifestyle section included questions on current smoking status (current smokers were defined as those who smoked one or more cigarettes per day for at least 6 months), alcohol intake (alcohol drinkers were defined as those who drank alcohol on average more than once a week within the last year), regular exercise (regular exercise was defined as any physical activity, such as walking, tai chi *etc.*, performed regularly for more than 20 min at a time each day, and excluding routine office and housework), physical examination, number of self-reported chronic diseases, hospitalization in the last year, body mass index (BMI), blood pressure (BP), and random capillary plasma glucose (RCPG). According to the Chinese BMI reference [36], subjects were categorized as “underweight” (<18.5), “normal weight” (18.5–23.9), “overweight” (24.0–27.9), and “obese” (≥28.0). The RCPG was measured in fresh capillary blood samples obtained from fingertip pricking by using glucose oxidase impregnated strips [37].

#### 2.2.3. Sociodemographic Characteristics

These measured the sociodemographic information, including age, marital status, educational level, employment status, health insurance, living arrangement, and number of children.

### 2.3. Ethics Statement

This study was approved by the Ethics Committee of Guangzhou Medical University, and written informed consent was obtained from all study participants before the investigation.

### 2.4. Statistical Analysis

Statistical analyses were performed using Statistical Package for Social Sciences (SPSS), version 13.0 (SPSS Inc., Chicago, IL, USA). Means and standard deviations (SD) were presented for continuous variables. Frequency and percentage were presented for categorical variables. We then categorized the fatigue in two categories: with and without fatigue. Chi square test or t-test were used to assess the differences in sociodemographic and health and lifestyle factors between subjects. At last, multivariate logistic regression models were employed to analyze the risk factors of fatigue. Both unadjusted and adjusted logistic regression models performed a forward stepwise selection strategy. Odds ratios (OR) with 95% confidence intervals (95% CI) were presented. Two tailed *p* values < 0.05 were considered statistically significant.

## 3. Results

There were 335 study participants who reported fatigue (CFS score ≥ 4), so the prevalence of fatigue was 28.9%. The mean CFS score was 2.56 (SD = 2.69) scores; 1.78 (SD = 2.13) scores for the PF and 0.77 (SD = 0.89) scores for MF. Univariate analyses of fatigue in relation to sociodemographic characteristics and health and lifestyle factors are presented in Table 1 and Table 2, respectively. 

**Table 1:** The sociodemographic characteristics associated with fatigue and its prevalence.

Variable	Fatigue	No Fatigue	Entire Sample	*p*
Age, years (m, SD)	62.86 ± 11.98	56.64 ± 9.32	58.44 ± 10.53	< 0.001 ***
Age, years (*n*, %)				< 0.001 ***
45–54	105 (19.7)	427 (80.3)	532 (45.9)	
55–64	94 (29.2)	228 (70.8)	322 (27.8)	
65–74	65 (34.6)	123 (65.4)	188 (16.3)	
≥75	71 (61.2)	45 (38.8)	116 (10.0)	
Marital status (*n*, %)				0.001 **
Single ^a^	30 (49.2)	31 (50.8)	61 (5.3)	
Married	305 (27.8)	792 (72.2)	1097 (94.7)	
Education level (*n*, %)				< 0.001 ***
No schooling	31 (41.3)	44 (58.7)	75 (6.5)	
Primary school	183 (35.5)	333 (64.5)	516 (44.5)	
Middle school	95 (21.9)	338 (78.1)	433 (37.4)	
High school or above	26 (19.4)	108 (80.6)	134 (11.6)	
Employment status (*n*, %)				< 0.001 ***
Employed	137 (21.5)	499 (78.5)	636 (54.9)	
Retired	80 (30.2)	185 (69.8)	265 (22.9)	
Unemployed	118 (45.9)	139 (54.1)	257 (22.2)	
Health insurance (*n*, %)				0.450
Yes	291 (28.6)	728 (71.4)	1019 (88.0)	
No	44 (31.7)	95 (68.3)	139 (12.0)	
Living arrangement (*n*, %)				0.002 **
Living alone	10 (45.5)	12 (54.5)	22 (1.9)	
Living with spouse only	46 (41.1)	66 (58.9)	112 (9.7)	
Living with children	279 (27.2)	745 (72.8)	1024 (88.4)	
Number of children (*n*, %)				<0.001 ***
≤1 ^b^	54 (20.4)	211 (79.6)	265 (22.9)	
2	152 (25.6)	442 (74.4)	594 (51.3)	
3	68 (36.0)	121 (64.0)	189 (16.3)	
≥4	61 (55.5)	49 (44.5)	110 (9.5)	
PF score (m, SD)	4.65 ± 1.50	0.61 ± 0.87	1.78 ± 2.13	< 0.001 ***
MF score (m, SD)	1.52 ± 1.08	0.47 ± 0.57	0.77 ± 0.89	< 0.001 ***
CFS score (m, SD)	6.17 ± 1.94	1.09 ± 1.07	2.56 ± 2.69	< 0.001 ***
Fatigue status (*n*, %)	335 (28.9)	823 (71.1)	1158 (100.0)	

Note: Data presented are mean ± SD or *n* (%); PF: Physical fatigue; MF: Mental fatigue; CFS: Chalder Fatigue Scale; ^a^ Single: unmarried, divorced or widowed; ^b^ Number of children: ≤1, include 6 men without children; * *p* < 0.05; ** *p* < 0.01; *** *p* < 0.001.

The epidemiological characteristics of fatigue and the sociodemographic characteristics are reported in Table 1. Chi square test showed that the prevalence of fatigue among the respondents had significant differences (*p* < 0.05) in age, marital status, education level, employment status, living arrangement, number of children, alcohol intake, regular physical exercise, physical examination, number of self-reported chronic disease, hospitalization in the last year, and BMI, while it had no significant difference (*p* > 0.05) in health insurance and current smoking status. Significant differences were observed for age, diastolic blood pressure (DBP), RCPG form *t*-test, but systolic blood pressure (SBP) did not.

**Table 2:** Health and lifestyle factors of participants with and without fatigue.

Variable	Fatigue	No Fatigue	Entire Sample	*p*
Current smoking (*n*, %)				0.260
Yes	120 (27.0)	324 (73.0)	444 (38.3)	
No	215 (30.1)	499 (69.9)	714 (61.7)	
Alcohol intake (*n*, %)				0.038 *
Yes	140 (26.0)	399 (74.0)	539 (46.5)	
No	195 (31.5)	424 (68.5)	619 (53.5)	
Regular exercise (*n*, %)				0.003 **
Yes	69 (22.3)	240 (77.7)	309 (26.7)	
No	266 (31.3)	583 (68.7)	849 (73.3)	
Physical examination (*n*, %)				<0.001 ***
Yes	169 (34.4)	322 (65.6)	491 (42.4)	
No	166 (24.9)	501 (75.1)	667 (57.6)	
No. of self-reported chronic disease (*n*, %)				<0.001 ***
0	143 (21.3)	529 (78.7)	672 (58.0)	
1	112 (33.4)	223 (66.6)	335 (28.9)	
≥2	80 (53.0)	71 (47.0)	151 (13.1)	
Hospitalization in the last year (*n*, %)				<0.001 ***
Yes	60 (51.7)	56 (48.3)	116 (10.0)	
No	275 (26.4)	767 (73.6)	1042 (90.0)	
BMI, kg/m^2^ (*n*, %)				0.011 *
Underweight	27 (47.4)	30 (52.6)	57 (4.9)	
Normal weight	155 (26.7)	426 (73.3)	581 (50.2)	
Overweight	118 (28.9)	290 (71.1)	408 (35.2)	
Obese	35 (31.3)	77 (68.8)	112 (9.7)	
SBP, mmHg (m, SD)	128.79 ± 16.02	128.02 ± 14.11	128.24 ± 14.69	0.444
DBP, mmHg (m, SD)	79.71 ± 8.93	80.96 ± 8.43	80.60 ± 8.59	0.025 *
RCPG, mmol/L (m, SD)	7.64 ± 2.60	7.22 ± 2.48	7.34 ± 2.52	0.011 *

Note: Data presented are mean ± SD or *n* (%); BMI, body mass index; SBP, systolic blood pressure; DBP, diastolic blood pressure; RCPG, random capillary plasma glucose. * *p* < 0.05; ** *p* < 0.01; *** *p* < 0.001.

All statistically significant variables in univariate analysis were applied to perform a stepwise logistic regression analysis, and the results are presented in Table 3. The results showed that age, marital status, employment status, children number, numbers of self-reported chronic diseases, whether hospitalized in the past year, and physical exercise were significantly associated with fatigue. The elderly group (≥75 years) were almost four-fold more likely to be fatigued (OR = 3.88, 95% CI 2.09–7.18) than the reference group (45–54 years). Men who maintained a single status (unmarried, divorced or widowed; OR = 1.94, 95% CI 1.04–3.62), unemployed status (OR = 1.68, 95% CI 1.16–2.43) and have four or more children (OR = 2.35, 95% CI 1.33–4.15) were more likely to express fatigue. Similarly, men who had more self-reported chronic diseases (≥2 chronic diseases: OR = 2.83, 95% CI 1.86–4.31; only one chronic disease: OR = 1.42, 95% CI 1.03–1.96), or had hospitalization in the past year (OR = 1.61, 95% CI 1.03–2.52) were more likely to experience fatigue. Additionally, it was shown that regular exercise was a protective factor against fatigue (OR = 0.46, 95% CI 0.32–0.65).

**Table 3:** Factors associated with fatigue from logistic regression models.

Variable	OR ^c^ (95% CI)	*p*	OR ^d^ (95% CI)	*p*
**Sociodemographic Characteristics**				
**Age**				
45–54	Reference			
55–64	1.68 (1.22–2.31)	0.002 **	1.46 (1.03–2.09)	0.035 *
65–74	2.15 (1.49–3.11)	<0.001 ***	1.88 (1.17–3.01)	0.009 **
≥75	6.42 (4.17–9.87)	<0.001 ***	3.88 (2.09–7.18)	< 0.001 ***
**Marital Status**				
Married	Reference			
Single ^a^	2.51 (1.50–4.22)	0.001 **	1.94 (1.04–3.62)	0.037 *
**Occupational status**				
Employed	Reference			
Retired	1.58 (1.14–2.18)	0.006 **	0.78 (0.50–1.19)	0.248
Unemployed	3.09 (2.27–4.21)	<0.001 ***	1.68 (1.16–2.43)	0.006 **
**Number of Children**				
≤1 ^b^	Reference			
2	1.34 (0.95–1.91)	0.099	1.72 (1.16–2.56)	0.007 **
3	2.20 (1.44–3.35)	<0.001 ***	2.07 (1.30–3.30)	0.002 **
≥4	4.86 (3.01–7.86)	<0.001 ***	2.35 (1.33–4.15)	0.003 **
**Health and Lifestyle Factors**				
**Physical Exercise**				
No	Reference			
Yes	0.63 (0.47–0.85)	0.003 **	0.46 (0.32–0.65)	< 0.001 ***
**Number of Self-Reported Chronic Disease**				
0	Reference			
1	1.86 (1.39–2.49)	< 0.001 ***	1.42 (1.03–1.96)	0.032 *
≥2	4.17 (2.88–6.03)	< 0.001 ***	2.83 (1.86–4.31)	< 0.001 ***
**Hospitalization in the Last Year**				
No	Reference			
Yes	2.99 (2.03–4.41)	< 0.001 ***	1.61 (1.03–2.52)	0.037 *

Note: OR = Odds ratio; 95% CI = 95% confidence interval; ^a^ Single: unmarried, divorced or widowed; ^b^ No. of children: ≤1, include 6 men without children; ^c^ Crude OR; ^d^ Adjusted for all other variables included in the table; * *p* < 0.05; ** *p* < 0.01; *** *p* < 0.001.

## 4. Discussion

### 4.1. Main Findings

Our study found that almost 30% of the participants in Chinese study population had experienced fatigue, and fatigue is associated with age, marital status, employment status, regular exercise, number of self-reported chronic diseases, number of individual’s children and hospitalization in the last year in middle-aged and elderly males.

### 4.2. Comparison with Previous Studies

The prevalence of fatigue was 28.9% among the present study population in the past month, which was similarly to previous studies [16,18,20,38], such as those of Van’T and colleagues who found that the overall prevalence of fatigue was 30.5% in Nijmegen (The Netherlands) [18]; Kocalevent and colleagues who demonstrated that 25.9% of male respondents reported fatigue in Germany [38] and Lu and colleagues who found that 27.2% of plateau soldiers suffered from fatigue in China [20]. However, other studies have reported different prevalences of fatigue in China. For example, a study conducted in Hong Kong estimated the overall incidence in males was 8.0% [15]. In the present study, the definition of fatigue used implied that brief periods, prolonged and chronic fatigue was included, in other words, there is no restriction in duration of fatigue, which may help explain the high prevalence. Other possible explanations for the finding include the study design and the heterogeneity of the study population.

Ricci and colleagues revealed that the incidence of fatigue among male workers in the US was 31.0% [21], and 32.65% of male medical staff experienced fatigue in China [25], and our study was also consistent with this, because 54.9% of the middle-aged and elderly males were still in the workforce in China. However, Peng and colleagues’ survey of Hubei Province’s male workers found that the estimated high prevalence of fatigue in male workers was 81.8% [22], and the possible explanations may be related to fatigue styles and assessment tools. In Peng’s study the Fatigue Assessment Instrument (FAI) was adopted to assess the subjective fatigue.

A longitudinal study for elder people at age 70, 78, and 85 found that fatigue prevalence was 29%, 53%, and 68%, respectively [39]. De Rekeneire and colleagues’ cohort study (a median follow-up of 111 months) found that the cumulative incidence of restricting fatigue was 31.1% for men and 42.1% for women among non-disabled community-living older adults [30]. The elder population was more likely to fatigue, but the adolescents didn't seem so. Prevalence of fatigue was found to range between 3.0% and 18.4% in adolescents [28,29]. Interestingly, the prevalence of fatigue in the current study was lower than for elderly elder, but higher than for young people. Generally, with aging, people are less energetic, with decreasing physiological functions and increased risk of diseases, which may further contribute to this phenomenon.

Although several studies found that there was a higher incidence of fatigue in women than in men [24,40], our previous study reported that the incidence of fatigue was 33.9% in middle-aged and elderly females [8], which is similar to the prevalence of middle-aged and elderly males in the present study. Another study also showed that the gender divergences of fatigue disappeared after adjusting for covariates [15]. Those findings indicating gender differences in fatigue thus remain controversial.

During the last decades many risk factors for fatigue have been identified, which range from sociodemographic, and lifestyle-related to mental status. In terms of sociodemographic characteristics, age and marital status has been reported to be associated with fatigue [24,41,42], Jason and colleagues found unemployed status as an independent risk factor for fatigue [2], and the relations may even be of reciprocal causation, ending with more severe fatigue [43]. In terms of health and lifestyle factors, regular exercise seemed to be a protective factor for fatigue [44,45]. Additionally, a large number of studies have demonstrated that chronic health problems were strongly associated with fatigue [46]. Mental health problems such as depression and anxiety may aggravate this phenomenon [5,47]. Similar to those studies above, we also found age, marital and occupational status, regular exercise and chronic health problems were associated with fatigue.

We also found the gender divergences existed in risk factors compared to our previous study, such as employment status, education level, regular exercise, and BMI. Interestingly, unemployment status in males rendered them more susceptible to fatigue, while this relation failed to be observed in female [8]. The gender differences of social role, physical structure and culture background may play important roles in it. In China, family economic support always comes from father, so unemployed males may experience more stress [48]. Whatever the cause, further study is needed to better understand the observed gender differences in the risk factors of fatigue.

In the present study, we also found the number of individuals’ children and hospitalization in the last year were associated with fatigue among men aged 45 and older. With increasing number of children, men suffer higher risk of fatigue, so we speculate that the heavy burden of raising offspring may create great pressure on the father and thus bring about fatigue, which suggests that sensible planning of the number of children may mitigate potential risk of fatigue. Additionally, previous studies demonstrated that health problems were negatively associated with fatigue [46,47]. Hospitalization experiences likely resulted in mental impairment and even caused lower quality of life [49], so hospitalization in the past year may lead to fatigue through physical and psychological pathways.

## 5. Limitations

There are also limitations of the study which should be addressed. Due to the cross-sectional nature of this study design, causal relationships could not be firmly deduced. Though all of the data was collected via face-to-face interviews, recall bias might be introduced. Some possible risk factors were neglected in the current study, such as sleep problems, depression, pain, duration of fatigue and cultural differences. Especially for the cultural differences factors which do exist, further promotion and application of the risk factors should be noted. Therefore, the causal relationships require a larger and more prospective cohort studies to confirm them.

## 6. Conclusions

A high prevalence of fatigue was shown among men aged 45 and older in China. The fatigue was associated with the individuals’ number of children, hospitalization in the last year, age, marital status, occupational status, regular physical exercise and number of self-reported chronic diseases. The determinants also suggested some directions for preventing fatigue, for example regular physical exercise. These findings may have implications in the risk assessment of fatigue and in helping to develop and implement targeted interventions in middle-aged and elderly males.

## Abbreviations

CFS: Chalder Fatigue Scale; PF: Physical fatigue; MF: Mental fatigue; BMI: Body mass index; BP: Blood pressure; SBP: Systolic blood pressure; DBP: Diastolic blood pressure; RCPG: Random capillary plasma glucose.
